# Genetic Parameters and Genome-Wide Association Studies of Quality Traits Characterised Using Imaging Technologies in Rainbow Trout, *Oncorhynchus mykiss*

**DOI:** 10.3389/fgene.2021.639223

**Published:** 2021-02-22

**Authors:** Carole Blay, Pierrick Haffray, Jérôme Bugeon, Jonathan D’Ambrosio, Nicolas Dechamp, Guylaine Collewet, Florian Enez, Vincent Petit, Xavier Cousin, Geneviève Corraze, Florence Phocas, Mathilde Dupont-Nivet

**Affiliations:** ^1^Université Paris-Saclay, INRAE, AgroParisTech, GABI, Jouy-en-Josas, France; ^2^SYSAAF, Station LPGP-INRAE, Rennes, France; ^3^INRAE, LPGP, Rennes, France; ^4^INRAE, OPAALE, Rennes, France; ^5^Les Sources de l’Avance, Pissos, France; ^6^MARBEC, University of Montpellier, CNRS, Ifremer, IRD, Palavas-les-Flots, France; ^7^INRAE, University of Pau & Pays Adour, E2S UPPA, UMR 1419 NuMéA, Saint-Pée-sur-Nivelle, France

**Keywords:** aquaculture, fat content, flesh colour, magnetic resonance imaging, Fatmeter, computer vision, genetic correlations, QTL

## Abstract

One of the top priorities of the aquaculture industry is the genetic improvement of economically important traits in fish, such as those related to processing and quality. However, the accuracy of genetic evaluations has been hindered by a lack of data on such traits from a sufficiently large population of animals. The objectives of this study were thus threefold: (i) to estimate genetic parameters of growth-, yield-, and quality-related traits in rainbow trout (*Oncorhynchus mykiss*) using three different phenotyping technologies [invasive and non-invasive: microwave-based, digital image analysis, and magnetic resonance imaging (MRI)], (ii) to detect quantitative trait loci (QTLs) associated with these traits, and (iii) to identify candidate genes present within these QTL regions. Our study collected data from 1,379 fish on growth, yield-related traits (body weight, condition coefficient, head yield, carcass yield, headless gutted carcass yield), and quality-related traits (total fat, percentage of fat in subcutaneous adipose tissue, percentage of fat in flesh, flesh colour); genotypic data were then obtained for all fish using the 57K SNP Axiom^®^ Trout Genotyping array. Heritability estimates for most of the 14 traits examined were moderate to strong, varying from 0.12 to 0.67. Most traits were clearly polygenic, but our genome-wide association studies (GWASs) identified two genomic regions on chromosome 8 that explained up to 10% of the genetic variance (cumulative effects of two QTLs) for several traits (weight, condition coefficient, subcutaneous and total fat content, carcass and headless gutted carcass yields). For flesh colour traits, six QTLs explained 1–4% of the genetic variance. Within these regions, we identified several genes (*htr1*, *gnpat*, *ephx1*, *bcmo1*, and *cyp2x*) that have been implicated in adipogenesis or carotenoid metabolism, and thus represent good candidates for further functional validation. Finally, of the three techniques used for phenotyping, MRI demonstrated particular promise for measurements of fat content and distribution, while the digital image analysis-based approach was very useful in quantifying colour-related traits. This work provides new insights that may aid the development of commercial breeding programmes in rainbow trout, specifically with regard to the genetic improvement of yield and flesh-quality traits as well as the use of invasive and/or non-invasive technologies to predict such traits.

## Introduction

Aquaculture produces high-quality animal protein that is low in saturated fat, ideal for satisfying increased global demand. Worldwide, one of the most commonly farmed salmonid species is rainbow trout (*Oncorhynchus mykiss*; 848 100 t in 2018, FAO, 2020). In France, fish farms dedicated to this species represent the majority of aquaculture operations; their production has historically been diversified into multiple target sizes, with the pan-size (250–350 g), and moderately large (>1 kg) or very large (>2.5 kg) individuals. Most of animals are female, diploid or triploid, and reared for the production of fillets, which are consumed fresh or smoked (Vandeputte et al., 2019). For both economic and environmental reasons, the main challenge in trout production is promoting efficient growth while maintaining the quality of the fillet. Growth is a major selection goal in all fish breeding programmes, but efforts have also been made to improve the quality of fillets and adapt the features of trout to market demands, such as the desire for larger fillets instead of whole pan-size trout. For processors, the main traits of interest are carcass yield, fillet yield, fillet trimming yield, fillet morphology and thickness, lipid content, and lipid distribution in the fillet. Consumers are mainly concerned with fillet or flesh quality, followed by fat content, colour, texture, and flavour (Rasmussen et al., 2001). For both parties, the heterogeneous distribution of lipids within a fish has major effects on product quality (Katikou et al., 2001). Specifically, subcutaneous adipose tissue is discarded during fillet trimming and is thus negatively linked to fillet yield, while the quantity of lipids in the flesh has strong effects on the sensory quality of fish, such as texture and flavour (Grigorakis, 2007), and its overall acceptability to consumers (Katikou et al., 2001).

To efficiently address these challenges, it is first necessary to have reliable data on processing and quality traits – especially regarding the quantification of fat and its distribution – from a large-enough population of animals to enable accurate genetic evaluation. To this end, various invasive and non-invasive technologies have been used in livestock to predict carcass composition (yields and fat deposition), but these approaches have been less commonly applied to the composition of meat (Scholz et al., 2015; Carabús et al., 2016).

To be useful, phenotyping methods must be accurate, fast, and affordable. Historically, flesh colour has been measured with the L^∗^a^∗^b^∗^ international system using a Minolta Chroma Meter, but advances in computer vision have offered intriguing possibilities for automation and for capturing heterogeneity in trout (Marty-Mahé et al., 2004; Kause et al., 2008). The use of ultrasound technology to predict carcass and fillet yields in live candidates limits human error from manual filleting or trimming, which can hinder estimates of additive genetic variation (Haffray et al., 2012, 2013a). To measure lipid content in fish flesh, chemical extraction methods such as the Soxhlet and Folch techniques provide accurate measurements, but are invasive, time-consuming, and prohibitively expensive for widespread use in breeding programmes. Computer vision involved the development of imaging systems using different kind of spectral sensor (digital cameras, infrared sensors, 3D scanners …), and image analysis algorithms based on technology like machine learning. Suitable alternatives may include indirect technologies based on microwaves (Douirin et al., 1998), nuclear magnetic resonance (Toussaint et al., 2002), or near-infrared spectroscopy, which are all less expensive, more rapid, and in some cases, portable (Gjerde and Martens, 1987). The accuracy of these measures may be lower than that of chemical analysis, but this may be offset by their lower cost and higher speed. However, these indirect methods have a critical shortcoming in that they do not provide information on the distribution of fat within the fillet, particularly the differentiation between the fat content of flesh and that of subcutaneous tissues, and the differentiation between muscle and fat within flesh. To estimate lipid content and its distribution, X-ray computed tomography (CT) and magnetic resonance imaging (MRI) can be applied to a representative cutlet and the results evaluated using image analysis, an approach that has already been used for the phenotypic estimation of fat content in fish (Gjerde and Martens, 1987; Kolstad et al., 2004; Marty-Mahé et al., 2004; Toussaint et al., 2005; Mathiassen et al., 2011; Collewet et al., 2013; Picaud et al., 2016). Digital imaging processing and the MRI are examples of computer vision systems (Fernandes et al., 2020). Thanks to the visual contrast between fat and muscle, subcutaneous and intramuscular fat can also be measured in images acquired with a charge-coupled device camera (Marty-Mahe et al., 2003) or with a desktop scanner (Kause et al., 2008; Collewet et al., 2013). However, this last method is restricted to fish with pigmented flesh and are invasive, since fish have to be sliced before analysis. For examples, digital imaging processing has been used to measure fat and pigment concentrations in live and slaughtered Atlantic salmon (Folkestad et al., 2008). Progress has been made in MRI, in particular the water-fat separation approach, for the non-invasive and rapid quantification of lipid content and distribution in fish. For example, MRI has been used to quantify and locate fat in Atlantic mackerel (Brix et al., 2009), to describe the heterogeneity of lipid distribution in the flesh of brown trout (Toussaint et al., 2005; Bock et al., 2017), and to characterise adipose tissues in shrimp (Sun et al., 2020).

The final step in improving trout breeding programmes is to incorporate knowledge of the genetic architecture underlying trait variation. To date, most previous studies aimed at elucidating the genetic parameters of growth-related and quality traits in rainbow trout have focused on traits of primary economic importance, such as carcass yield, fillet yield, fat content in muscle, and fillet colour (Gjerde and Schaeffer, 1989; Haffray et al., 2012, 2014; Lhorente et al., 2019; Hu et al., 2020); two such selection programmes reported realised genetic gain in the range of 0.5–1% for fat in the muscle (Quillet et al., 2005) and carcass and fillet yields, as predicted by ultrasound technology (Vandeputte et al., 2019). However, the genetic architecture of yield traits is still poorly understood, and almost no attention has been paid to quality traits such as flesh colour or fat distribution. One major area of progress, though, has been the recent development of the Affymetrix^®^ Axiom^®^ 57K Trout SNP array (Palti et al., 2015) and the publication of the rainbow trout reference genome assembly (GenBank assembly Accession GCA_002163495, RefSeq assembly accession GCF_002163495), which have provided the genomic tools necessary for genome-wide association studies (GWASs) aimed at the detection of quantitative trait loci (QTLs) for traits of interest. In rainbow trout, GWAS has been used to identify significant QTLs associated with such traits of interest as growth or disease resistance (Laghari et al., 2014; Kocmarek et al., 2015; Liu et al., 2015; Abdelrahman et al., 2017; Ashton et al., 2017; Vallejo et al., 2017; Fraslin et al., 2018; Reis Neto et al., 2019; Ali et al., 2020b). Recently, studies have begun to focus on quality traits like fillet yield (Gonzalez-Pena et al., 2016; Al-Tobasei et al., 2017), muscle yield (Salem et al., 2018), skinned and trimmed fillet yield (Al-Tobasei et al., 2020), shear force and fillet firmness (Al-Tobasei et al., 2017, 2020; Ali et al., 2019), fillet whiteness as determined by L^∗^a^∗^b^∗^ characteristics (Al-Tobasei et al., 2017), and intramuscular fat content and moisture (Ali et al., 2020a). However, these studies were carried out on two experimental lines of trout that had been previously selected for growth or for resistance to *Flavobacterium psychrophilum* by the NCCCWA breeding program in the United States (Leeds et al., 2016) and in China (Hu et al., 2020). It remains to be seen whether the results can be generalised across fish of different ages or among commercial lines with different genetic backgrounds.

In addition to major quality traits, other traits that merit further investigation include fillet colour with respect to individual L^∗^a^∗^b^∗^ characteristics (lightness, red, and yellow, respectively), and not just whiteness; the potential to increase fillet fat content; and the ability to limit trimming losses of dorsal and ventral flesh (which are essentially composed of fatty tissues). A more-thorough understanding of the genetic architecture of these traits could prove useful for the refinement of breeding objectives, particularly in balancing the economic trade-off between different traits.

Within this context, the main objectives of this study were to (1) estimate and compare the genetic parameters of growth-, yield- (body weight, condition coefficient, head yield, carcass yield, headless gutted carcass yield) and quality-related traits (total fat, percentage of fat in subcutaneous adipose tissue, percentage of fat in flesh, flesh colour) in a commercially selected population of rainbow trout using three different phenotyping methods (microwave-based, digital image analysis, and MRI), (2) detect QTLs associated with these traits through GWAS, using the 57K SNP Axiom^®^ Trout Genotyping array, and (3) identify candidate genes present within these QTL regions.

## Materials and Methods

### Ethics Statement

This study used fin clips collected by the breeding company “Les sources de l’Avance” as part of their commercial breeding programme. Rearing was carried out in compliance with Directive 98/58/CE on the protection of animals kept for farming purposes and Directive 2010-63-EU on the protection of animals used for scientific purposes. Data were collected from sacrificed animals and thus the experiment did not require approval from an Ethics Committee, in accordance with Article 1.5 of Directive 2010-63-EU.

### Fish Production and Trait Recording

All measured traits and parameters of the rearing environment were defined according to the ATOL (Animal Trait Ontology for Livestock) (Table 1) and EOL (Environment Ontology for Livestock) databases, available on the ATOL website^1^. The fish came from a commercially selected line from the Sources de l’Avance breeding company (Pissos, France), a subsidiary of Aqualande Group. In December 2016, 831 rainbow trout families were created using a partly factorial mating design. Through 10 factorial crosses, 84 dams were crossed with 99 neomales (sex-reversed females used as sires). A piece of fin was sampled for each parent for the purpose of DNA extraction. Half-sib families from each dam were incubated in eleven troughs containing five trays divided into two compartments. At 15 days post-fertilisation (dpf) corresponding to eyed stage, similar numbers of eggs (*N* = 700) from each dam were mixed together after fecundation assessment and were reared in 12 separate fiberglass tanks (capacity ± 200 L; density between 46 and 68 kg/m^3^; feeding management ad libitum 5–6%). At 94 dpf (fish about 0.5–1 g), the 12 groups were moved to 4 concrete raceways divided into three compartments (1 group by compartment) (capacity ± 7 m^3^; density between 5 and 20 kg/m^3^ adjusting by water volume variation; feeding management ad libitum 4–6%). At 163 dpf, juvenile fish (33 g mean weight) were vaccinated against *Yersinia ruckeri* and *Aeromonas salmonicida*, and transferred to the “Viviers de la Hountine” trout farm (Belin-Béliet, France), located 12 km downstream of the hatchery, for further growth. The 12 groups were mixed and fish were reared in concrete raceways until the end of experiment (density 40–100 kg/m^3^ adjusting by water volume variation). Fish were fed to satiation (EOL_0001740) using extruded commercial feed: Neo start (17% lipids) and Neo CDC (23% lipids) (Le Gouessant, Lamballe, France) during the first stage, then Extra CDC AQL P/F/G25 (25–30% lipids) (Le Gouessant, Lamballe, France) and Viva Pro 9F NAT29 (30% lipids) (Aqualia, Arue, France) until the end of the experiment. In both farms, rearing units were supplied with flow through river water (EOL_0001571). The water temperature varied from 3 to 20°C (EOL_0000034, EOL_0000176, and EOL_0000246) and oxygen concentration was not limited (>80% saturation: EOL_0000186).

**TABLE 1:** Phenotypic descriptions of the 14 traits measured, with mean, standard error of the mean, minimum, maximum, coefficient of variation (CV), and sample size (*N*).

Trait	Description	Mean	SE	Minimum	Maximum	CV	*N*
BW	Body weight (g)	2142.93	12.05	658	3363	22	1,509
K	Fulton coefficient *K* = (*BW*(g) × 100/BL^3^ (cm))	1.38	0.003	0.99	1.80	9	1,509
Fat	% Fat with Fatmeter	13.88	0.07	4.3	20.3	18	1,505
Head%	Head yield (Head% = head weight/BW × 100)	10.84	0.02	8.15	15.58	8	1,509
Carc%	Carcass yield (Carc% = (BW − viscera weight)/ BW × 100	91.47	0.03	87.19	94.87	1	1,503
HGCarc%	Headless gutted carcass yield (HGCarc% = (BW − (Head weight + viscera weight))/BW × 100)	80.64	0.03	75.36	84.63	2	1,503
MRI_F_sc%	Percentage of subcutaneous fat in whole steak	27.01	0.07	16.42	37.74	10	1,350
MRI_F%	Percentage of fat in whole steak	26.25	0.08	16.01	34.84	11	1,351
MRI_F_F%	Percentage of fat flesh in whole steak	12.54	0.05	6.61	19.83	16	1,351
L_flesh	Flesh colour Luminosity	49.62	0.04	44.90	55.99	3	1,509
a_flesh	Flesh colour a redness	35.66	0.05	26.13	40.38	5	1,509
b_flesh	Flesh colour b yellowness	43.68	0.05	31.48	49.96	4	1,509
Adip%	Percentage of subcutaneous adipose tissue in whole steak	26.21	0.07	14.86	37.70	11	1,509
Myo%	Percentage of myosepta in whole steak	0.76	0.02	0.01	7.20	91	1,509

Fish rearing followed classical practices. Because measurements were performed only on slaughtered fish from a commercial population, there was no need to consult an ethics committee. At 469 dpf, fish were individually tagged with RFID transponders and their DNA was collected through a fin sample that was preserved in 95% ethanol for parentage assignment.

At the end of the growing period in April 2018, 1,510 fish were randomly sampled and divided into four sub-groups. Each sub-group was treated the same way: three days of fasting (EOL_0001739), followed by live transportation by truck to the Aqualande processing plant (Roquefort, France, 50 km from the growing farm), where they were slaughtered and processed for analysis. Because RNA was to be sampled for future expression analyses, fish were humanely killed by a blow to the head and bled by cutting the gills in ice water, in accordance with good animal slaughtering practices. Post-mortem data collection and processing were accomplished as quickly as possible to ensure data accuracy.

**FIGURE 1:**
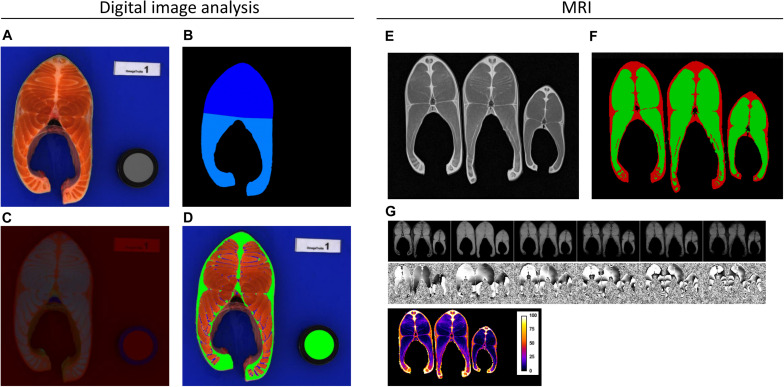
Examples of images obtained using digital image analysis **(A)** Steak prior to image analysis. **(B)** Dorsal and ventral segmentation. **(C)** Image for L*a*b analysis. **(D)** Differentiation among adipose tissue (green), myosepta (blue), and flesh (red). using MRI **(E)** ‘Spin echo’ MRI image. **(F)** Differentiation between subcutaneous fat (red) and flesh (green). **(G)** Module and phase images, and map of lipid percentage in a whole steak.

Data collection was performed between 503 and 506 dpf. The traits that were quantified included body weight (BW, ATOL_0000351), fork length (BL, ATOL_0001658), head weight (HeadW, ATOL_0001545), headless gutted carcass weight (HGCarcW, ATOL_0002260), and viscera weight (ViscW, ATOL_0002258). These traits were then combined to create the following synthetic traits and yields (see Table 1): Fulton’s coefficient of condition, calculated as *K* = BW(g) × 100/BL^3^ (cm) (ATOL_0001653); headless gutted carcass yield (HGCarc%, ATOL_0002261); head yield (Head%, ATOL_0005561); and gutted carcass yield (Carc%, ATOL_0000548). The total fat content in muscle (Fat, ATOL_0001663) was recorded using a Fish Torry Fat-meter^®^ as described in Douirin et al. (1998); the probe was placed on the left side and the minimum value was recorded. For each fish, one cross-sectional steak was sampled, in front of the dorsal fin, and photographed using a digital camera (Canon EOS 1000 D 10 M Pixels, pixel size 74 μm); the camera was fixed to a copy stand and used in concert with a shooting tent (Literoom Photoflex©) to avoid specular reflection. Steaks were then packed in individual plastic bags and kept frozen at −20°C until MRI analysis for logistic reasons. The place where MRI was carried out was 570 km away from the slaughtering place and it was not possible to carry out MRI just after the slaughtering operations. Digital pictures were analysed using the method described by Marty-Mahe et al. (2003), with some modifications. Images were first converted in the L^∗^a^∗^b^∗^ and HLS colour space, then, using Visilog 7.3 for Windows©, colour image segmentation was performed to quantify the area of the steak (ATOL_0005553), peripheral fat tissue (ATOL_0005562, including red muscle), and myosepta (Figure 1A). Briefly the steak was separated from the background using a manually set threshold on the red channel followed by a manual correction and separation of the dorsal and ventral parts. In the steak, flesh was manually thresholded on the S channel, then the subcutaneous fat tissue was deduced as the pixels in the steak that did not belong to the flesh. Finally the myosepta in the flesh were segmented using a black tophat of the S channel and a fixed threshold. As recommended by the CIE 1976 (Robertson, 1977), the colour of flesh (ATOL_0001017), including myosepta, was expressed in the L^∗^a^∗^b^∗^ system, which represents its lightness, redness, and yellowness, respectively. Tissue area was normalised by the entire surface area of the steak (SS) to obtain the following yields (see Table 1): myosepta percentage (Myo%) and subcutaneous fat percentage (Adip%).

Two different protocols were used for the acquisition of MRI images; each corresponded to virtual slices of 5-mm thickness acquired in the middle of the steak. To quantify subcutaneous fat, a “spin-echo” image was used, which had a resolution of 0.75 mm × 0.75 mm in the image plane and provided a good contrast between flesh and lipid (Figure 1E). Subcutaneous fat area was then estimated (Figure 1F) using an automatic image analysis scheme as described in Collewet et al. (2013). The steak was separated from the background using an automatic thresholding. The kidney was excluded from the steak. The flesh was defined as the set of voxels with an intensity smaller than a threshold which was chosen manually. It was the same for each steak in one level, but varied between levels to cope for the variation of the signal intensity with the position due to inevitable MRI imperfections. Small fat insertion in the muscle were attributed to flesh and small areas of muscle included in the fat near the dorsal fin were attributed to fat. Subcutaneous fat was deduced as the set of voxels in the steak that did not belong to the flesh. The fat content in flesh was estimated using the protocol described in Picaud et al. (2016), which is based on “gradient echo” imaging (module and phase) at 6 different “echo times,” thus allowing cartographic estimation of lipid percentage (Figure 1G). The spatial resolution for these images was 1.5 mm × 1.5 mm in the image plane. In both protocols, steaks were placed 15 by 15 in the MRI. A total of 45 steaks were analysed at a time, arranged 3 by 3 in 5 levels, one on top of the other. The total acquisition time was 16 min. Twenty-four hours prior to MRI analysis, the steaks were placed at the temperature used for imaging, 4°C. These images were then used to compute three traits: subcutaneous fat percentage (MRI_F_sc%), calculated as the area of subcutaneous fat divided by steak surface area (SS); total fat content (MRI_F%), and fat content in flesh (MRI_F_F%), which were both calculated as a percentage of the whole steak and computed from the lipid percentage images (see Table 1).

In total, 14 variables were evaluated in this study (see Table 1): body weight (BW); Fulton coefficient (K); head yield (Head%); carcass yield (Carc%); headless gutted carcass yield (HGCarc%); percentage of total fat measured with Fat-meter (Fat) and MRI (MRI_F%); subcutaneous fat percentage measured by digital image analysis (Adip%) and by MRI, which included dorsal and ventral fat that is typically trimmed (MRI_F_sc%); flesh fat percentage measured by MRI (MRI_F_F%) and myosepta percentage calculated by digital image analysis (Myo%); and three flesh colour variables measured by vision (L_flesh, a_flesh, and b_flesh).

### Genotyping

DNA samples from all 1,510 offspring, 99 sires, and 84 dams were sent to the INRAE genotyping platform Gentyane (Clermont-Ferrand, France) for DNA extraction and genotyping. Following DNA quality control, 1,680 of the 1,693 samples (183 parents and 1,497 offspring) remained; these were genotyped for 57,501 SNPs using the 57K SNP Axiom^®^ Trout Genotyping array from Thermo Fisher (Palti et al., 2015). The number of progeny per sire varied from 5 to 39, with an average of 17, and from 6 to 39 per dam, with an average of 21.

Quality control of SNPs was performed in several steps, as described in D’Ambrosio et al. (2019), in particular to remove SNPs with probe polymorphism and multiple locations in the genome. The following filters were applied: call rate higher than 0.97, deviation from Hardy-Weinberg equilibrium with a *p*-value > 0.0001, and a minor allele frequency (MAF) higher than 0.05. In total, 29,652 SNPs were retained for the analysis. Samples in which less than 90% of SNPs were genotyped were removed from the dataset. All missing SNP genotypes of the remaining individuals were imputed using FImpute software 2.0 (Sargolzaei et al., 2014).

The final dataset therefore contained 1,379 fish with phenotypes and genotypes (for 29,652 SNPs) which were subjected to GWAS analysis.

### Estimation of Genetic Parameters

Heritability (*h*2) and phenotypic and genetic correlations (*r*p and *r*g, respectively) were estimated for all traits using the restricted maximum likelihood method (AIREML) in BLUPF90 software (Misztal et al., 2002). For each trait, the model adopted to describe its performance was:

$$Y_{i}=\mu +u_{i}+bBW_{i}+e_{i}$$

where *Y*_*i*_ is the performance of the *i*th animal, μ is the overall mean of the population, *u*_*i*_ is the additive effect of the *i*th animal, *b* is the regression coefficient of *Y* on *BW*, *BW*_*i*_ is the weight of the *i*th individual, and *e*_*i*_ is the residual random error term. The pedigree considered was constituted of 17,235 animals over nine generations. The vector *u*_*i*_ corresponded to the breeding values of 17,235 individuals related through the pedigree relationship matrix A. Maternal effects were not significant and thus not included in the final models for any trait. All fish were female and reared in the same raceway since 163 dpf, so that no fixed effect was included in the model.

Heritabilities were estimated using univariate analyses. Heritability estimates were calculated as additive genetic variance ($\sigma_{u}^{2}$) divided by the total phenotypic variance ($\sigma_{p}^{2}$). Genetic correlations were estimated using the model above with bivariate analyses as

$$rg\frac{cov\left(u1,u2\right)}{\sqrt{(\sigma_{u1}^{2}\times \sigma_{u2)}^{2}}}$$

where cov(*u*1, *u*2) is the additive genetic covariance between trait 1 and trait 2, and $\sigma_{u1}^{2}$ and $\sigma_{u2}^{2}$ are the additive genetic variance of trait 1 and 2.

### Genome-Wide Association Study and QTL Detection

A Bayesian Stochastic Search Variable Selection approach BayesCπ (Habier et al., 2011) was implemented in BESSiE software (version 1.0) (Boerner and Tier, 2016) to perform GWASs.

In the BayesCπ model, only a certain proportion of SNPs (π) are thought to have a non-zero effect on the phenotype. The marker effects are estimated through an MCMC algorithm that considers a mixture of markers, of which proportion π have effects that follow a normal distribution *N*(0, $\sigma_{a}^{2}$) and proportion 1 – π have zero effect. The general model used is:

$$Y_{i}\mu +bBW_{i}+\sum_{j1}^{n}\delta_{jk}a_{j}g_{ij}+\varepsilon_{ik}$$

with *Y* the phenotype observed for the *i*th individual, μ the overall mean in the population, *b* the regression coefficient of *Y* on *BW*; *n* the total number of SNPs in the analysis, *a*_*j*_ the additive effect of the reference allele for the *j*th SNP, with genotype *g*_*ij*_ for individual *i* (coded as 0, 1, or 2); and ε_*ik*_the residual effect for the *i*th individual in the *k*th iteration. The vector of residual effects is normally and independently distributed, $\varepsilon N\left(0,\text{I}\sigma_{e}^{2}\right)$, with $\sigma_{e}^{2}$ the residual variance.

At each cycle *k*, the decision to include SNP *j* in the model depended on the indicator variable δ*_*jk*_*: if δ*_*jk*_* was equal to 1, the effect of SNP j was estimated as *a*_*j*_, while if δ*_*jk*_* was equal to 0, no effect was estimated. This indicator variable was sampled from a binomial distribution with a probability π that δ*_*jk*_* was equal to 1 (i.e., the SNP has a non-zero effect) and a probability 1 − π that δ*_*jk*_* was equal to 0. The proportion 1 − π was sampled from a beta distribution, B(α, β), in which α was set as the total number of markers (*n* = 29,652) and β was set at 300, so that approximately 1% of the SNPs were included in the model at each cycle. A total of 200,000 cycles were used, with a burn-in period of 5,000 cycles; Gibbs sampling was performed every 20 cycles and saved for further analysis. In order to check the convergence, the MCMC algorithm was initiated three times with three different seeds for the random number generator. Convergence was assessed by visual inspection of plots of the posterior density of genetic and residual variances and by high correlations (*r* > 0.99) between the genomic estimated breeding values (GEBVs) estimated from the different seeds of the MCMC algorithm.

The degree of association between each SNP and a given phenotype was assessed using the Bayes Factor (BF): $\text{BF}=\frac{P_{i}/\left(1-P_{i}\right)}{\pi /\left(1-\pi \right)}$, where *P*_*i*_ is the probability that the *i*th SNP has a non-zero effect.

Following Kass and Raftery (1995), evidence for QTLs were evaluated based on calculations of 2 × ln(BF); specifically, values higher than or equal to 6 were considered evidence for the existence of the QTL. As proposed by Michenet et al. (2016), a credibility interval was constructed around the peak SNP which integrated within the QTL region all SNPs with 2 × ln(BF) ≥ 3 that were located close to the peak SNP using a sliding window of 1 Mb on both sides of the peak SNP. If 6 ≤ 2 × ln(BF) < 8 for a peak, the QTL was considered putative, and was characterised only if it explained at least 1% of the genetic variance for the trait. Instead, values of 2 × ln(BF) that were equal to or higher than 8 were considered to provide strong evidence of a QTL.

All candidate genes that were located within the confidence or credibility intervals estimated using the BayesCπ approach are listed in Supplementary Table 1, with annotation from the NCBI *O. mykiss* genome assembly release 100 (GCF_002163495.1), and gene symbols from Lallias et al. (2020). Genes that were close to the peak or that had been previously highlighted in the literature were subjected to further discussion.

## Results

In this study, 1,510 rainbow trout were phenotyped for multiple traits, which were then investigated using descriptive statistics and the estimation of genetic parameters. Cross-sectional steaks of 1,351 fish were subjected to MRI analysis. Finally, a GWAS analysis was carried out that included genotypes from 1,379 individuals. Descriptive statistics (mean, standard error, and number of fish used in the analysis) for phenotypic traits are given in Table 1. Briefly, the mean weight at slaughter was 2,143 g. Carcass yield and headless gutted carcass yield were 91 and 81%, respectively. Subcutaneous fat content was measured using two different protocols: no mean differences were detected in the percentage of subcutaneous fat (26.2%) as assessed by MRI and by digital image analysis but the phenotypic correlation was moderate (0.54). Percentage of fat was 13.9% estimated with the Fatmeter and 26.5% with MRI with phenotypic correlation of 0.77. Percentage of fat in the flesh was 12.5% when measured by MRI. The coefficient of variation for fat-related traits was calculated as 18% using the Fatmeter, between 10 and 16% for the MRI method, and between 11 and 91% for digital image analysis. The coefficient of variation was lower than 10% for all colour and yield traits.

### Heritability and Correlation Estimates

**FIGURE 2:**
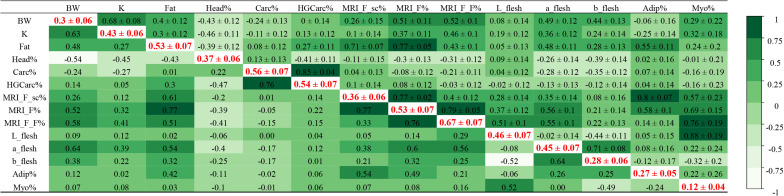
Genetic parameters in rainbow trout. Heritabilities (± standard error) in bold red on the diagonal (with body weight as a covariate), genetic correlations (± standard error) in the upper triangle, phenotypic correlations in the lower triangle.

The heritability estimates of yield and quality traits, and their phenotypic and genetic correlations, are given in Figure 2.

In our study, heritability estimates of all traits were moderate to high, which suggests that these traits can be improved through selective breeding (Figure 2). Among similar phenotypes, heritability values were higher for traits characterised using MRI technology than with digital image analysis. The highest heritability was estimated for the quantity of lipids in flesh detected using MRI (*h*2 _(*MRI_F_F%*)_ = 0.67 ± 0.07), while the lowest was calculated for the percentage of myosepta in flesh as determined by digital image analysis (*h*2 _(*Myo%*)_ = 0.12 ± 0.04). However, we identified a strong genetic correlation between these two phenotypes (*r*g = 0.76 ± 0.19), which are both associated with the lipid content of flesh. Similarly, the heritability of subcutaneous adipose tissue was higher when it was quantified using MRI technology than with digital image analysis (*h*2 _(*MRI_F_sc%*)_ = 0.36 ± 0.06 and *h*2 _(*Adip%*)_ = 0.27 ± 0.05), and both traits were strongly genetically correlated (*r*g = 0.80 ± 0.07). The genetic correlation between the amount of lipids in flesh (MRI_F_F%) and both measurements of subcutaneous adipose tissue (MRI_F_sc% and Adip%) was low (*r*g = 0.40 ± 0.11 and 0.14 ± 0.14, respectively). The estimated heritability of fat content as measured with a Fatmeter was the same as the heritability estimate for total fat in the steak as quantified using MRI technology, *h*2 = 0.53 ± 0.07, with a strong genetic correlation between the two traits (*r*g = 0.77 ± 0.05 with MRI_F%). Stronger genetic correlations were detected between Fat (measured by Fatmeter) and both measurements of subcutaneous adipose tissue (*r*g = 0.71 ± 0.07 with MRI_F_sc% and *r*g = 0.55 ± 0.11 with Adip%) than between Fat (using Fatmeter) and lipids in the flesh (MRI_F_F% using MRI; *r*g = 0.43 ± 0.10) or myosepta (Myo% using digital image analysis; *r*g = 0.24 ± 0.2).

Headless gutted carcass yield (HGCarc%), which is used as an indirect predictor of fillet yield (Haffray et al., 2014), was highly heritable (0.54 ± 0.07), but was not correlated to steak adiposity as measured by MRI (*r*g = −0.03 ± 0.12 with MRI_F_F%).

Regarding colour traits, heritability estimates for L_flesh, a_flesh, and b_flesh were 0.46 ± 0.07, 0.45 ± 0.07, and 0.28 ± 0.06, respectively. We detected the strongest genetic correlations between a_flesh and both measurements of fat (Fat, *r*g = 0.48 ± 0.11, and MRI_F%, *r*g = 0.56 ± 0.10). Our results indicated a stronger genetic correlation between colour traits (L^∗^a^∗^b flesh) and the quantity of lipids in flesh (MRI_F_F%) than with subcutaneous adipose tissue (MRI_F_sc% and Adip%), with the highest correlation estimated for a_flesh and the lowest for b_flesh. L_flesh was strongly correlated with Myo% (*r*g = 0.88 ± 0.19) but moderately with MRI_F_F% (*r*g = 0.51 ± 0.10), and was only slightly correlated with the total quantity of lipids (Fat or MRI_F%).

### Quantitative Trait Loci Detection

Using the genotypes from 29,652 SNPs, GWAS analyses were performed for the fourteen traits of interest. Several QTLs were detected for yield and quality traits, and their characteristics are given in Table 2.

**TABLE 2:** Summary of the characteristics of QTLs detected using BCπ methods. Chr: Chromosome; 2 × ln(BF): twice the natural logarithm of the Bayes Factor; MAF: minor allele frequency.

Trait	Chr.	Peak SNP	SNP peak position (Mb)	2 × ln(BF)	MAF	QTLstart position (Mb)	QTLend position (Mb)	% of variance explained by QTL	% of variance explained by peak SNP
BW	8	AX-89920070	14.03	6.80	0.48	13.32	15.10	2.36	0.57
BW	22	AX-89955188	8.30	8.27	0.37	6.77	8.41	2.06	0.87
K	7	AX-89955974	24.39	7.61	0.35	24.12	25.05	1.38	0.63
K	8	AX-89962581	13.32	9.41	0.36	11.62	14.14	2.48	1.41
K	8	AX-89920477	18.81	8.65	0.5	17.83	21.61	7.83	1.30
K	13	AX-89958775	16.39	8.41	0.49	15.02	18.75	1.81	0.62
K	16	AX-89936112	36.75	9.15	0.44	36.75	37.56	1.2	0.76
Fat	5	AX-89940295	8.17	10.19	0.16	7.75	8.99	1.31	0.85
Fat	8	AX-89962581	13.32	9.29	0.36	13.32	15.10	2.88	1.43
Fat	8	AX-89969787	18.99	7.58	0.44	18.51	25.79	5.7	0.78
Head%	3	AX-89972128	51.09	7.13	0.24	49.21	51.35	1.56	0.43
Head%	22	AX-89973440	4.93	7.66	0.31	4.46	5.03	1.25	0.55
Carc%	4	AX-89966085	3.07	10.56	0.48	1.98	3.07	1.11	1.06
Carc%	7	AX-89961082	7.52	7.72	0.41	7.52	8.62	1.8	0.63
Carc%	8	AX-89962581	13.32	6.32	0.36	12.91	13.46	0.76	0.33
Carc%	8	AX-89959583	21.42	8.71	0.42	18.95	23.82	3.27	0.81
HGCarc%	8	AX-89959583	21.42	10.05	0.42	18.51	23.56	4.10	1.27
MRI_F_sc%	3	AX-89966492	73.73	7.48	0.36	73.26	74.45	1.31	0.57
MRI_F_sc%	8	AX-89962581	13.32	11.91	0.36	13.32	15.10	5.41	3.27
MRI_F%	3	AX-89966492	73.73	7.87	0.36	73.26	74.45	2.08	0.67
MRI_F%	8	AX-89962581	13.32	9.40	0.36	13.32	15.10	4.6	1.61
MRI_F_F%	2	AX-89955617	47.18	8.29	0.37	46.77	47.73	1.37	0.70
MRI_F_F%	12	AX-89947824	74.45	10.18	0.28	74.26	75.25	1.18	0.98
L_flesh	11	AX-89935698	63.48	6.97	0.34	61.24	63.91	1.87	0.46
L_flesh	17	AX-89966607	65.97	8.83	0.40	64.49	66.29	4.22	1.45
a_flesh	24	AX-89945908	10.45	7.09	0.36	9.85	10.45	1.14	0.49
b_flesh	6	AX-89956277	61.53	7.89	0.23	61.53	62.53	1.62	0.69
b_flesh	6	AX-89950758	64.69	6.41	0.49	63.54	64.69	0.68	0.36
b_flesh	13	AX-89919579	21.95	10.51	0.43	21.34	22.93	3	1.91
Adip%	8	AX-89920070	14.03	9.48	0.48	12.45	15.10	4.04	1.82
Adip%	8	AX-89933187	18.51	7.27	0.41	17.25	18.99	1.36	0.60

*The percentage of genetic variance explained by a QTL was calculated as the sum of the variance, from the BCπ model, of all SNPs in the credibility interval of the QTL. The credibility interval (QTL start/QTL end) was constructed around the peak SNP and contained all SNPs with 2 × ln(BF) ≥ 3 that were located close to the peak SNP using a sliding window of 1 Mb on both sides of the peak SNP.*

**FIGURE 3:**
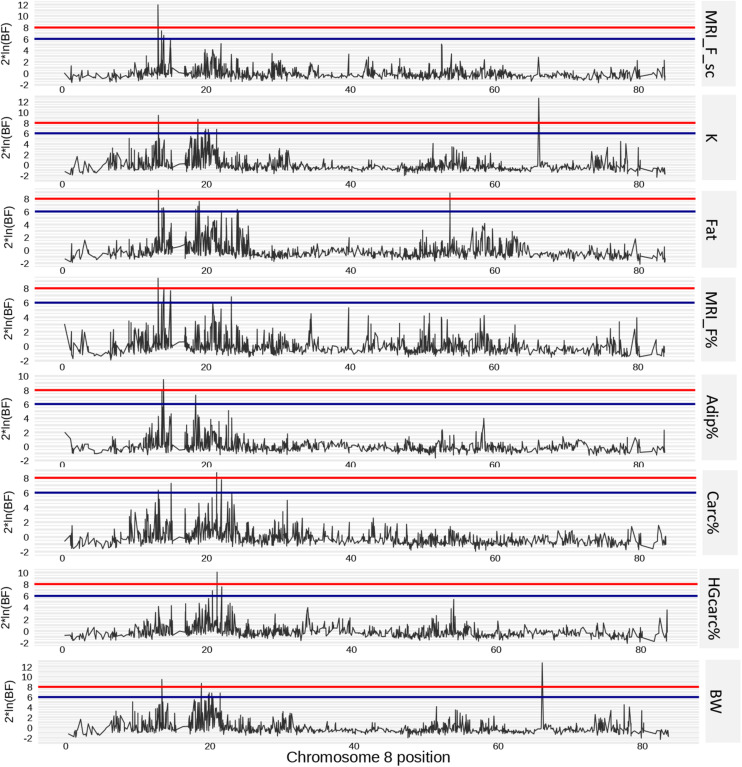
Positions of SNP markers associated with phenotypes of interest on chromosome 8. Putative (2 × ln(BF) = 6) and strong (2 × ln(BF) = 8) effect thresholds are represented by blue and red horizontal lines, respectively.

On Omy8, one QTL was identified in a first region located between 11.62 and 15.10 Mb. We found strong evidence that this region had effects on five traits: K, Fat, MRI_F_sc%, MRI_F%, and Adip%. Depending on the trait, this region explained between 2.48 and 5.41% of the genetic variance and had a credibility interval between 1,777 and 2,654 kb wide. There was also weaker evidence that this region contained QTLs for BW and Carc% that explained 2.36 and 0.76% of their respective genetic variance. A second region on Omy8 (located between 17.25 and 25.79 Mb) was identified as another QTL for K, Fat, HGCarc%, Carc%, and Adip%. This region explained a higher percentage of genetic variance – 7.83, 5.7, 4.1, 3.27, and 1.36%, respectively – and had credibility intervals that ranged from 1,739 to 7,279 kb in size (Figure 3).

Strong evidence was found for a QTL on Omy22 with effects on BW, which explained 2.06% of the genetic variance of this trait.

For colour traits, one QTL was detected on Omy17, between 64.49 and 66.29 Mb, that explained 4.2% of the genetic variance in L_flesh. Three QTLs were found for b_flesh (Table 2), of which one on Omy13 explained 3.0% of the genetic variance and two relatively close QTLs on Omy6 together explained 2.3% of the genetic variance in this trait.

No QTL was found that explained more than 2% of the genetic variance in a_flesh, Head%, or MRI_F_F%, which suggests that these traits have a very polygenic architecture affected by multiple loci with small effects in this population of rainbow trout.

### Identification of Candidate Genes

The candidate genes identified within the different QTL regions are detailed in Supplementary Table 1.

In total, 18 genes were positioned between two SNP peaks (13.32 and 14.10 Mb) on chromosome 8, which together corresponding to seven QTLs spanning from 11.62 to 15.10 Mb (see Supplementary Table 1). The peak SNP for K, Fat, MRI_F_sc%, and MRI_F%, at 13.32 Mb, was located in the intergenic region between the *bub1* and *htr1e* genes. *Bub1* codes for the mitotic checkpoint serine/threonine protein kinase and *htr1e* for the 5-hydroxytryptamine receptor 1E (Seeley et al., 1999; Tsukahara et al., 2010). The peak for BW and Adip% was located on Omy8 in the intergenic region between the *bach2b* (BTB and CNC homolog 1) gene and the *map3k7* (mitogen-activated protein kinase kinase kinase 7) gene, at 14.10 Mb.

The second QTL region on Omy8 spanned from 17.25 to 25.79 Mb. Between the three peaks for Adip%, Fat, and K, there were 17 genes with annotation and two uncharacterised proteins. Two of the peak SNPs were located in intergenic regions between, respectively, *vgll2a* (vestigial-like family member 2a) and *rfx6* (regulatory factor X6), and *gnpat* (glyceronephosphate O-acyltransferase) and *ext1c* [exostoses (multiple) 1c], while the third peak SNP was located within the *s22a2* (solute carrier family 22 member 2) gene. The two peaks for Carc% and HGCarc% on Omy8 (at 21.42 Mb) were detected in the intergenic region between the *ephx1* (epoxide hydrolase 1) gene and the *srp9* (signal recognition particle 9) gene.

Within the QTL associated with L_flesh on Omy17, we found 34 annotated genes. The peak SNP for this QTL was located in the intergenic region between *rplp2l* (ribosomal protein, large P2,-like) and *adora2b* (adenosine A2b receptor). For b_flesh, the peak SNP on Omy13 was located within the *kif16ba* (kinesin family member 16Ba) gene at 21.95 Mb. Two peak SNPs for QTLs on Omy6 were located in *mical2a* (microtubule-associated monooxygenase, calponin and LIM domain containing 2a) and *tcf25* (transcription factor 25), respectively. Other candidate genes were also identified within the QTL regions associated with these traits, but at more of a distance from the peaks. For example, *bcmo1* (beta,beta-carotene 15,15′-dioxygenase) was located in a QTL for b_flesh on Omy6, and is known to be involved in carotenoid metabolism. Likewise, *dkk3a* (dickkopf WNT signalling pathway inhibitor 3a) and *bola3* (bolA family member 3) were located in a QTL for b_flesh on Omy6; both are known to be involved in adipogenesis (Christodoulides et al., 2006; Huang et al., 2019; Tajima et al., 2019). Two genes within the CYP family, which comprises a large number of genes that encode P450 enzymes, were identified in two different QTLs for b_flesh. The gene *cyp2x7* (cytochrome P450, family 2, subfamily X, polypeptide 7) was located within a QTL on Omy6 while *cyp2x9* (cytochrome P450, family 2, subfamily X, polypeptide 9), coding for a vitamin D 25-hydroxylase-like enzyme (Cheng et al., 2004; Uno et al., 2012), was found in the QTL on Omy13.

## Discussion

### Phenotype Measurements and Implications for Selective Breeding

Image and acceptance of aquaculture products, as well as the profitability of such products for growers and processors, can be strongly shaped by phenotypic traits such as growth rate, carcass and fillet yields, flesh colour, and the lipid content of fillets. This last trait can also influence technical properties related to fillet processing (Sodeland et al., 2013), fillet colour and texture (Johansson et al., 2000; Mørkøre et al., 2001; Lefevre et al., 2015), and the organoleptic quality and nutritional value of flesh (Zheng et al., 2016). For the purposes of genetic selection, the accuracy of measurement of a phenotype is of primary importance, but this must be balanced against the cost of measurement. For this reason, studies such as this one, which explicitly test different phenotyping methods, are crucial to the development of selection programmes. Here, we compared three technologies (microwave-based, digital image analysis, and MRI) to characterise the quantity and/or distribution of lipids in rainbow trout flesh. Microwave-based methods (Fatmeter) have been used by French trout breeders since they were first made available for use (Douirin et al., 1998). Initial studies based on this approach estimated that the heritability of fat was between 0.36 and 0.72, depending on the position of the probe on the fish and the design of the experiment (Chevassus et al., 2002; Quillet et al., 2005). In our study, the heritability of this trait was 0.53, within the range from the literature and similar to results reported from a previous generation of the same population (Haffray et al., 2018). Likewise, heritability values estimated for the percentage of myosepta (Myo%; 0.12) and adipose tissue (Adip%; 0.27) as determined by digital image analysis, and subcutaneous fat as determined by MRI (*h*2 > 0.50), were close to values that had been previously estimated for another population from another breeding company (Haffray et al., 2013b). We did not include tank effect in our model. Since fish were reared in common since 163 days, it is likely that common environmental effects were not significant. However, we cannot exclude that they were significant, which could lead to overestimation of the heritabilities. In our study, traits related to adipose tissue (subcutaneous and flesh) that were quantified with MRI technology had higher heritabilities (0.36–0.67) than those measured with digital image analysis (0.27–0.12), which could indicate a higher degree of precision for MRI phenotyping. For the subcutaneous fat, the voxel size in MRI is much greater than the pixel size in the digital image analysis-based method (750 or 1500 μm vs. 74 μm), which would seem to promote higher variability in the former with respect to the latter. Thanks to the flexibility of the MRI technique, the contrast between muscle and fat could be optimised unlike for a digital camera. For the fat content in flesh, the relatively large size of the MRI voxel is not a potential disadvantage since we are interested in the mean of the fat content over the flesh. MRI measurement integrates fat content over the thickness of the voxel, which could make it less sensitive to the position of the cut than digital image analysis. Moreover, MRI is able to detect low fat content. Indeed, the variation of the MRI signal with the echo time, which is used to estimate the fat content, is theorically lost in the noise only below 2% of fat with the signal to noise ratio observed in our experiment (around 60). On the other hand, the digital image analysis approach is based on the segmentation of each myosepta as this tissue is a site of fat deposition. But this measure is more indirect than MRI. Indeed it is based on the hypothesis that the myosepta area is a proxy of the fat content. On the other hand, the MRI signal is directly linked to the actual quantity of lipids within each voxel. Thanks to the difference of frequencies between water and lipids signals, they can be separated. The amplitude of the lipid signal is proportional to the quantity of lipids, and the quantification does not require a segmentation process but just the calculation of the mean intensity of the voxels. Moreover the quality of myosepta segmentation is based on flesh colour not flesh composition as the MRI signal and flesh fat is not only located in the myosepta. So even if the resolution of digital image analysis is higher than MRI image the information include in MRI image is more precise to quantify subcutaneous and flesh fat tissue. Another interesting finding of our study was that the quantity of lipids estimated by Fatmeter was more genetically correlated to the amount of subcutaneous adipose tissue (0.55; 0.71) than to the amount of fat in flesh (0.24; 0.43) as measured by vision or MRI technologies, respectively. This suggests that selection for fat based on measurements from a Fatmeter might have a greater effect on subcutaneous adipose tissue than on lipid content in the flesh; this effect might be particularly pronounced in large fish, as previous work reported a strong indirect correlation with lipids in the flesh for pan-size trout (Quillet et al., 2005). Another study of the Fatmeter reported findings about the correlation between fat meter values and subcutaneous tissue thickness: the fat in the flesh was less likely to be correctly measured with thicker subcutaneous tissue (Clerjon and Bugeon, 2006). Compared to digital image analysis and MRI, measurement with a Fatmeter is rapid, inexpensive, and non-destructive. More work is clearly needed to further evaluate what kind of fat is truly selected for using a Fatmeter-based approach. From a breeder’s perspective, this distinction is essential, as the quantity of lipids is a key parameter but also the distribution of lipids within tissues can have critical effects on the quality of the product. The quantity of lipids in flesh has strong effects on sensory perception and gustation, while subcutaneous adipose tissue is discarded during fillet processing, meaning that its abundance is directly linked to fillet yield. As trout fillet is much leaner than that of Atlantic salmon, one breeding objective is likely to be an increase in or the maintenance of lipid content in the fillet in order to facilitate salting and smoking, two preparations that are popular with consumers. Instead, subcutaneous adipose tissue is less desirable, and breeders might seek to decrease this trait in order to limit production losses during dorsal and ventral trimming. Our results suggest that, in designing selection programmes to increase fat in the fillet, breeders face a choice: inexpensive phenotyping using a Fatmeter, with the potential for increases in subcutaneous fat in large trout as a collateral effect, or more expensive, but more precise, MRI-based phenotyping. Because the heritability of MRI-based traits was higher than that of Fatmeter-based traits, an ideal genetic programme would incorporate MRI-based traits measured directly from breeding candidates. However, if MRI phenotyping is unavailable for candidates, it is possible that direct measurement with a Fatmeter would be preferable to sib selection based on MRI. Combining MRI and genomic selection should be the most efficient strategy.

The main advantage of MRI technology is that it enables accurate quantification of the lipid distribution in flesh and subcutaneous fat in a large number of samples. Compared to digital image analysis, for which each steak needs to be carefully checked to prevent contamination by scales or blood, the MRI method used here is also faster (one cutlet every 1–2 min). In this respect, MRI also has an advantage over X-ray computer tomography; in two recent reports, the maximum number of whole pigs that could be analysed per day using this method was 15 (Scholz et al., 2015; Carabús et al., 2016). The major disadvantage of MRI technology is the high price of the equipment, but the access to apparatus dedicated to animals or agro-industry could allow potential application for many areas and production systems. Access may be further improved through the ability to freeze cutlets for later MRI analysis using centralised and specialised equipment, as was done in this study. Thanks to the freezing stage, the cutlets can be transported to the MRI, and analyzing the cutlets 15 by 15 reduces the acquisition time which turns down to a cost comparable to a chemical extraction. The number of cutlets analysed simultaneously could also be increased, and the cost decreased, by acquiring the images at room temperature, which would release the space taken by the refrigerating system. Such an approach may improve the speed of the analysis, reduce costs, and facilitate the development of MRI-based high-throughput phenotyping. Before this approach can be applied at a large scale, however, the impact of freezing on fat measurement by MRI must be evaluated, as a freezing effect has been reported for X-ray computer tomography (Gjerde and Martens, 1987). Additionally, studies should be conducted to assess the differences between live and dead fish.

The approach based on digital image analysis was able to differentiate subcutaneous adipose and myosepta, and was unique among the three methods we evaluated in the ability to simultaneously evaluate the colour of flesh, which is genetically correlated to the quantity of lipids in flesh (Bugeon et al., 2010). However, the percentage of myosepta as measured by digital image analysis had a large coefficient of variation (91%) and low estimated heritability (0.12) in this population (in contrast to the results of Kause et al. (2008), who reported estimates of *h*2 above 0.5 for percent lipid stripe in a different population reared in a very different environment). It thus appears that myosepta measurements using this technique were less accurate than those of subcutaneous fat. Despite this drawback, digital image analysis certainly represents a good compromise between the accuracy of measurement and the price for subcutaneous tissue and colour. The main disadvantage is that this method is destructive, and thus, information from siblings must be used to evaluate breeding candidates. Nevertheless, its use as part of a well-designed genomic selection programme may offer advantages that offset the lower efficacy of sib selection for such traits.

Our study mainly targeted the development of muscle and adipose tissues. However, processing yields can be strongly affected by bony tissues, for which alternative technologies such as X-ray tomography (Ceballos-Francisco et al., 2020) or Dual-energy X-ray absorptiometry (Ndiaye et al., 2020) may be useful in characterising development or in predicting the relative phosphorus composition of carcasses.

In salmonids, flesh colouration is an important quality parameter, with consumers having marked preferences for red-coloured products (Skonberg et al., 1998). The perceptible colouration of many seafoods is due to carotenoid pigmentation (Shahidi et al., 1998). The addition of carotenoids (astaxanthin and canthaxanthin) to fish diets is common and contributes to flesh pigmentation in salmonids (Storebakken and No, 1992; Bjerken, 2000). Interestingly, we report here estimates of heritability for flesh colour parameters (0.26–0.46) that are on the higher end of the range reported in the literature (from 0.16 to 0.46 for muscle or fillet colour traits recorded using the Roche scoring card for rainbow trout and Atlantic salmon (Gjerde and Schaeffer, 1989; Iwamoto et al., 1990; Quinton et al., 2005; Powell et al., 2008). We also found higher heritability for flesh colour than a previous study using the same colour system (L^∗^a^∗^b^∗^) in Atlantic salmon (0.05–0.20) (Norris and Cunningham, 2004), but our results were similar to what has been previously published for rainbow trout (Kause et al., 2008; Haffray et al., 2018). This confirms that there is definite potential for selection for the improvement of colour traits as recorded by image analysis.

Because pigments are liposoluble, colour parameters (L^∗^a^∗^b^∗^) are also correlated to the fat content of muscle (Nutreco and Matforsk, 1998). The a^∗^ (red) and b^∗^ (yellow) qualities are pure colour components whose values depend only on flesh pigmentation. Instead, L^∗^ (luminosity) is affected by the quantity of lipids: the fattier the flesh, the thicker the myosepta (corresponding to white striations in the flesh), which leads to higher luminosity (Bugeon et al., 2010). Because of this, if we select L_flesh, the quantity of fat in flesh will be moderately selected (*r*g = 0.51) without the use of MRI. The high genetic correlation between Myo% and L_flesh (*r*g = 0.88 ± 0.19), and the higher heritability and accuracy of L_flesh compared to Myo%, suggests that selection on L_flesh is possible, and this approach could replace one based on MRI phenotypes for the selection of lipids in flesh. It thus seems that flesh colour can be effectively improved by selection; however, future studies should explore the stability of the genetic correlation between L_flesh and Myo% at different pigmentation levels.

Together, these results reveal considerable genetic potential that can be exploited by selection. Both of the imaging technologies used here (vision and MRI) have the ability to inform decisions about the genetic improvement of quality based on tissue differentiation. Improvement in the digital image analysis especially for the segmentation of the myosepta could be a way to quantify more precisely muscle fat. Beside the classical segmentation techniques, the development of deep learning methods could allow a better segmentation on low contrast images (Fernandes et al., 2020). However, these techniques require the training of a CNN (convolutional neural network) like U-Net (Falk et al., 2019) with manually annotated images (ground truth). The quality of the manual annotation being a key point for the prediction by the CNN. The construction of such manually annotate image databases is a quite tedious task and the precision difficult to obtain on thin tissue such as the myosepta.

Going beyond the quantity and distribution of fat in tissues, another interesting selection goal is the nutritional quality of fat (content of healthy *n* − 3 long chain polyunsaturated fatty acids in fillets). A previous study revealed the potential of selective breeding to increase the levels of *n* − 3 LC PUFA in salmon (Horn et al., 2018), but similar studies in trout are lacking.

### Newly Identified QTLs for Muscle Fat Content and Production Traits in Trout

In this study, we identified new QTLs associated with production and flesh-quality traits in rainbow trout. However, our work is consistent with previous reports (Gonzalez-Pena et al., 2016; Salem et al., 2018; Ali et al., 2020a) in characterising the vast majority of these traits as polygenic.

Previous studies have identified QTLs on chromosomes 14 and 16 with strong effects on muscle yield (explaining up to 28.4% of the additive genetic variance; Salem et al., 2018), regions on chromosome 9 associated with fillet yield and fillet weight (explaining less than 1.5% of the genetic variance), and a region on chromosome 5 linked with body weight (Gonzalez-Pena et al., 2016). Additionally, an older study detected QTLs for body weight and condition factor on linkage groups RT-9 (corresponding to Omy12) and RT-27 (corresponding to Omy2) (Wringe et al., 2010). More recently, QTLs for muscle fat and moisture content were identified on chromosomes 19 and 29 in rainbow trout (Ali et al., 2019, 2020a). In farmed Atlantic salmon, significant QTLs were identified on chromosomes 13, 18, and 20 with effects on growth and yield-related traits (Tsai et al., 2015) and on chromosome 9 and 10 for fat content (Sodeland et al., 2013). A recent GWAS performed in Atlantic salmon reported that growth-related traits are controlled by variants with small effects (Yoshida et al., 2017). Our study did not detect any of these previously described QTLs; instead, all QTLs identified here for yields and muscle fat were in different regions of the trout genome. Our GWAS analysis identified genomic regions that explained up to 10% of the genetic variance for several traits (BW; K; Fat; Carc%; HGCarc%; MRI_F_sc%; MRI_F; Adip%), with particularly strong evidence for two regions on chromosome 8. Specifically, these regions had detectable effects on total fat content as measured by both Fatmeter and MRI (Fat and MRI_F%, respectively) and subcutaneous adipose tissue as quantified by digital image analysis (Adip%) and MRI (MRI_F_sc%). However, this region was not associated with fat located in the flesh, regardless of the measurement approach used (MRI: MRI_F_F%, or vision: Myo%). Potential candidate genes located in these regions are discussed below.

For flesh colour traits, our study detected two QTLs on Omy6, relatively close to each other. This chromosome corresponds to chromosome 26 in Atlantic salmon, where two previous studies have also detected a QTL for flesh colour (flesh pigment content measured as astaxanthin level; Tsai et al., 2015; Helgeland et al., 2019).

Overall, the heterogeneity in the QTL results makes it difficult to compare our results to those of previous studies. In general, comparisons of QTL regions across studies have suggested that QTLs tend to be population-specific, due to the polygenic nature of traits, possible genotype-by-environment interactions, and different histories of genetic selection. However, inconsistencies among studies might also arise from other factors: the use of different GWAS approaches; large variations in population size, breeding design, pedigree structure, and data collection; and differences in the SNP arrays – and consequently, marker density – used across studies.

### Candidate Genes Implicated in Adipogenesis and Carotenoid Processes

In a 2018 study, Salem et al. highlighted a candidate gene for muscle yield that was involved in citrate synthase activity. Interestingly, none of the candidate genes identified here were linked to this function. Instead, a single region on Omy8 was linked with K, Fat, MRI_F_sc%, MRI_F%, BW, Adip%, Carc%, and HGCarc%, which suggests the existence of a common mechanism underlying these traits. In this QTL region (one of two we identified on Omy8), two Htr receptors (*htr1b* and *htr1e*) were located near the peak SNP and may represent good functional candidate genes. Serotonin, or 5-hydroxytryptamine, is a biogenic monoamine that can function as both a neurotransmitter and a hormone. The 5-hydroxytryptamine (Htr) receptor is known to be involved in many important biological functions, including appetite, homeostasis, and gastrointestinal functions (Gershon, 2013); inflammatory responses (Mauler et al., 2016); regulation of lipid metabolism, together with peroxisome proliferator-activated receptors (PPAR) (Waku et al., 2010; Khademi et al., 2019); or the regulation of body weight or obesity (Crane et al., 2015; Oh et al., 2015; Wyler et al., 2017). Serotonin has also been shown to influence both lipid and glucose metabolism directly by suppressing lipolysis and glucose uptake in primary adipose cells (Hansson et al., 2016). Specifically, the serotonin 1E receptor gene (*htr1e*) has been linked with feed intake in rainbow trout (Callet et al., 2018) and in rats (Hayes and Covasa, 2006). A recent study in pigs reported that intramuscular adipocytes showed dose-dependent serotonin stimulation, which then resulted in decreased fat accumulation in mature adipocytes (Tada et al., 2020). A GWAS in sheep linked a SNP close to *htr1e* with internal fat content (Hernández-Montiel et al., 2020). In Yan Yellow cattle, RNA-seq analysis revealed a positive role for another serotonin receptor, *htr2a*, in adipogenesis (Yun et al., 2018). In humans, genetic studies have reported that polymorphisms in Htr genes (for example, *HTR1A*, *HTR1Dβ*, and *HTR2A*) are associated with obesity (Gorwood et al., 2002; Levitan et al., 2006; Compan et al., 2012; Oh et al., 2015).

In the first region on Omy8, we also identified two other genes, *bach2* and *map3k7*. The transcriptional regulator bach2 is a key regulator of adaptive immunity (Afzali et al., 2017), while *map3k7* (also called transformed growth factor activated kinase-1), a member of the serine/threonine protein kinase family, is an essential component of the MAP kinase signal transduction pathway and plays a role in cell proliferation by controlling diverse cell functions, including transcription, apoptosis, and immune response (Yamaguchi et al., 1995; Zou et al., 2019). The second QTL region on Omy8 contained two genes linked to lipid metabolism. The first, *gnpat*, codes for the dihydroxyacetone phosphate acyltransferase protein involved in glycerophospholipid metabolism, which is part of membrane lipid metabolism (Ofman et al., 2001). The second, *ephx1*, may play a role in the metabolism of endogenous lipids such as epoxide-containing fatty acids (Decker et al., 2012). Based on their effects and their known associations with lipid metabolism, the genes *htr1*, *gnpat*, and *ephx1* all represent good functional candidates.

With respect to flesh colour, a QTL region on Omy6 associated with the trait b_flesh contained the gene *bcmo1* (beta,beta-carotene 15,15′-dioxygenase-like), which is known to play a part in carotenoid metabolism. Bcmo1 participates in an early step of retinoic acid synthesis, the conversion of carotenoids of vegetable origin into retinal [which is then converted into retinoic acid (RA) by Raldh]. There is abundant evidence in the literature that RA is able to block adipogenesis. In addition, RA triggers the activation of the retinoid X receptor RXR, which forms heterodimers with PPAR (a major regulator of lipid metabolism) in order to activate target genes (Hessel et al., 2007). Unfortunately, the characterisation of carotenoid oxygenase in aquatic species has been very limited. A study in scallops identified the carotenoid oxygenase gene *PyBCO-like 1* as the key gene for carotenoid colouration in muscle (Li et al., 2019), while another study on flesh colour in salmon highlighted two other genes involved in carotenoid metabolism: *bco1* (beta-carotene oxygenase 1) and *bcol1* (beta-carotene oxygenase 1-like) (Helgeland et al., 2019).

Our GWAS analysis of flesh colour traits highlighted a region on Omy6 that contains two genes, *dkk3* and *bola3*, known to be involved in adipogenesis. This finding was consistent with previous work reporting a correlation between colour parameters and the fat content of muscle (Nutreco and Matforsk, 1998). In particular, *dkk3* was recently reported to induce adipogenesis and angiogenesis in 3 T3-L1 preadipocytes (Christodoulides et al., 2006; Huang et al., 2019), and, via GWAS, it was identified as a strong candidate gene for fat deposition in pigs (Liu et al., 2019). Instead, *bola3* encodes a protein that facilitates production of iron-sulfur (Fe-S) clusters, which then play a key role in the maturation of lipoate-containing 2-oxoacid dehydrogenases and the creation of respiratory chain complexes in the mitochondria. A recent study revealed that the fat-specific deletion of *bola3* results in a defect in Fe-S cluster formation that significantly reduces mitochondrial lipoylation and fuel oxidation in brown adipose tissue, leading to glucose intolerance and obesity (Tajima et al., 2019).

Using GWAS, we were able to detect a number of markers with significant associations for traits linked with fat, yield, and flesh colour. Before this information can be used for the purpose of selection, further studies are required to identify the most-valuable SNPs and the most-desirable alleles, which can then be incorporated in the design of breeding programmes.

## Conclusion

In summary, this work provides new insights into the genetics of traits associated with yield and flesh quality in rainbow trout which can be applied to commercial breeding. First, we confirmed the applicability of MRI technology for the selection of fat content in flesh and digital image analysis technology for the selection of flesh colour. Our results confirm that yield and quality traits are polygenic, and most are moderately to strongly heritable in rainbow trout. Despite this polygenic architecture, we were able to link these traits with several genomic regions that explained 1–8% of the genetic variance. A region on Omy8 was identified as having particularly strong effects on condition coefficient, fat content, and headless gutted carcass yield. When we investigated the genes within these regions, we identified several (*htr1*, *gnpat*, *ephx1*, *bcmo1*, and *cyp2x*) that have been implicated in adipogenesis or carotenoid metabolism. These represent good candidates for further functional validation to uncover the biological mechanisms underlying variation in yield and flesh quality in rainbow trout.

## Data Availability Statement

The datasets presented in this article are not readily available because the data that support the findings of this study are available from the breeding company “Aqualande” and restrictions apply to the availability of these data, which were used under license for the current study, and so are not publicly available. The data can be made available for reproduction of the results on request via a material transfer agreement and with permission of the breeding company “Aqualande.” Requests to access the datasets should be directed to mathilde.dupont-nivet@inrae.fr and vincent.petit2@wanadoo.fr.

## Ethics Statement

Ethical review and approval was not required for the animal study because this study used fin clips collected by the breeding company “Les sources de l’Avance” as part of their commercial breeding programme. Rearing was carried out in compliance with Directive 98/58/CE on the protection of animals kept for farming purposes and Directive 2010-63-EU on the protection of animals used for scientific purposes. The data were collected from sacrificed animals and thus the experiment did not require approval from an ethics committee, in accordance with Article 1.5 of Directive 2010-63-EU.

## Author Contributions

PH, MD-N, GeC, and VP contributed to the conception and design of the study. CB, JB, ND, GeC, FE, and MD-N collected the data. GuC carried out the MRI data collection. CB and FE organised the database. CB, JB, JD’A, ND, and FE performed the data analysis. CB, ND, FP, and MD-N collaborated on the design of the statistical methodology and interpretation of the results. FP and MD-N supervised the data analysis. CB wrote the first draft of the manuscript, with additional sections written by JB and XC. MD-N, PH, and FP contributed to the manuscript revision. All authors read and approved the submitted version.

## Conflict of Interest

The authors declare that the research was conducted in the absence of any commercial or financial relationships that could be construed as a potential conflict of interest.

## Footnotes

1 https://www.atol-ontology.com/en/
